# Roles of Reactive Oxygen Species in Biological Behaviors of Prostate Cancer

**DOI:** 10.1155/2020/1269624

**Published:** 2020-09-29

**Authors:** Chenglin Han, Zilong Wang, Yingkun Xu, Shuxiao Chen, Yuqing Han, Lin Li, Muwen Wang, Xunbo Jin

**Affiliations:** ^1^Department of Urology, Shandong Provincial Hospital, Cheeloo College of Medicine, Shandong University, Jinan, Shandong 250021, China; ^2^Department of Vascular Surgery, Shandong Provincial Hospital, Cheeloo College of Medicine, Shandong University, Jinan, Shandong 250021, China; ^3^Department of Radiology, Shandong Provincial Hospital, Cheeloo College of Medicine, Shandong University, Jinan, Shandong 250021, China; ^4^Department of Orthopedics, Shandong Provincial Hospital, Cheeloo College of Medicine, Shandong University, Jinan, Shandong 250021, China; ^5^Department of Urology, Shandong Provincial Hospital Affiliated to Shandong First Medical University, Jinan, Shandong 250021, China

## Abstract

Prostate cancer (PCa), known as a heterogenous disease, has a high incidence and mortality rate around the world and seriously threatens public health. As an inevitable by-product of cellular metabolism, reactive oxygen species (ROS) exhibit beneficial effects by regulating signaling cascades and homeostasis. More and more evidence highlights that PCa is closely associated with age, and high levels of ROS are driven through activation of several signaling pathways with age, which facilitate the initiation, development, and progression of PCa. Nevertheless, excessive amounts of ROS result in harmful effects, such as genotoxicity and cell death. On the other hand, PCa cells adaptively upregulate antioxidant genes to detoxify from ROS, suggesting that a subtle balance of intracellular ROS levels is required for cancer cell functions. The current review discusses the generation and biological roles of ROS in PCa and provides new strategies based on the regulation of ROS for the treatment of PCa.

## 1. Introduction

PCa has the highest prevalence for males in Europe as well as America and is also the second leading cause of cancer-related deaths for males [1]. In the year 2020, approximately 1,920,000 new cases of PCa are expected to be diagnosed, of which 33,000 may die [2]. The incidence of PCa has increased in recent years, notably in developing countries, which is strongly associated with economic development and lifestyle [2–5]. Multiple processes are involved in malignant transformation of prostate cells, initiating as prostatic intraepithelial neoplasia (PIN) followed by localized PCa. The early stages of PCa progression are treated by radical prostatectomy and localized radiation [1]. Once these therapies fail, the standard treatment for late-stage PCa is aimed at preventing androgen binding to AR (androgen deprivation therapy, ADT) or inhibiting AR activity directly (antiandrogens). This strategy comes from the fact that the primary prostate tumor is mostly made up of Androgen Receptor-positive (AR+) cancer cells, which are initially androgen-dependent. Despite responding to ATD during the first 14-20 months, almost all patients acquire resistance and progress into castration-resistant prostate cancer (CRPC) with primary metastasis of the lymph nodes or bones [6]; it is often fatal, and the overall survival (OS) is relatively low. Therefore, the treatment of PCa remains a formidable challenge and enigma.

ROS are a class of highly reactive, oxygen-containing molecules mainly including superoxide anion, hydrogen peroxide, hydroxyl radicals, and singlet oxygen [7], which cannot be detected directly in human specimens due to their short half-lives [8]. Hydroxyl radical (OH^−^) is the most unstable and reacts fleetly with adjacent biomolecules. Additionally, hydrogen peroxide (H2O2), as the major species of ROS, can cross the cell membranes and exert effects beyond the cell limits [9]. Intracellular ROS levels are tightly dependent on the various synthesis and degradation pathways. Maintenance of ROS at physiological levels is crucial to redox regulation involving repair, survival, and differentiation [7, 10]. However, either excessive generation of ROS or a decrease in the free radical scavenging system may increase ROS levels, thus inducing oxidative stress that acts as an etiological factor for wide varieties of pathologies, such as diabetes, myocardial injury, and cancer [4, 10]. As two-faced molecules, ROS have either beneficial or deleterious effects on PCa cells. Many experimental and clinical results have demonstrated that higher levels of ROS, particularly free radicals, can cause oxidative damages in DNA, proteins, and lipids, further contributing to the pathogenesis and the progression of PCa [11, 12]. Thus, it is reasonable to anticipate that the use of antioxidants has the potential to prevent and treat prostate carcinogenesis by eliminating ROS and oxidative stress. Besides, further accumulation of ROS could disturb normal cellular processes, eventually resulting in cell death [13, 14].

This current review aims to focus on proposed mechanisms by which ROS either promote or inhibit the progression of PCa and provides clues for anticancer therapies based on redox regulation. With respect to the extensive pleiotropy of ROS, the emerging field of redox medicine has received increasing attention in recent years. Therefore, further studies are required to elucidate the relationship between ROS and PCa.

## 2. Sources of Intracellular ROS in PCa

**Figure 1:**
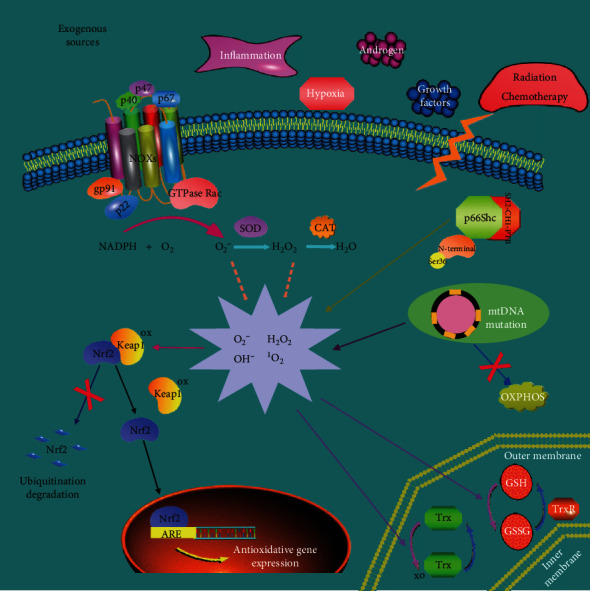
ROS generation and increased antioxidants in PCa cells. The generation of ROS is mainly dependent on both exogenous and endogenous sources. Exogenous sources comprise hypoxia, growth factors, androgen, inflammation, radiation, and chemotherapy; endogenous sources of ROS mainly include mitochondrial dysfunction, the activity of NADPH oxidases, and p66Shc. When ROS levels rise, PCa cells can responsively modulate Keap1/Nrf2/ARE axis and upregulate antioxidants to prevent their accumulation and deleterious actions. Increased antioxidants involve SOD, CAT, Trx, and GSH, whereas antioxidant defenses cannot neutralize elevated ROS, thus disrupting the redox homeostasis. Eventually, a new state called as oxidative stress arises. OXPHOS: oxidative phosphorylation; Keap1: Kelch-like ECH-associated protein 1; ARE: antioxidant responsive element; NOXs: NADPH oxidases; SOD: superoxide dismutase; CAT: catalase; Trx: thioredoxin; GSH: glutathione. Dash arrows indicate the class of ROS, while filled arrows indicate direct or indirect actions.

Both endogenous and exogenous sources promote the generation of intracellular ROS. Higher levels of basal ROS in PCa cells result from mitochondria dysfunction, increased p66Shc, glucose metabolism (Warburg effect), and the activation of enzymes including NADPH oxidases, xanthine oxidases, and cytochrome P450 [15]. In the following paragraphs, we especially pay attention to mitochondria dysfunction, NADPH oxidases, and p66Shc activation, which are significant contributors of endogenous ROS in PCa [16]. On the other hand, ROS generation is also driven in response to extracellular stimuli, such as hypoxia, growth factors, androgen, and inflammation (Figure 1). Growth factors activate the small RhoGTPase K-ras downstream to elevate intracellular superoxide levels through mitochondria or NADPH oxidases [17].

### 2.1. Mitochondria Dysfunction

Mitochondrial electron transport chain (ETC) composed of complex I, III, and IV induces oxidative phosphorylation (OXPHOS) to produce ATP with a by-product ROS generation due to inevitable electron leakage to O_2_, which is identified as the major endogenous source of ROS [18]. It is well documented that mitochondrial DNA (mtDNA), double-stranded circular DNA, is resident in the mitochondrial matrix encased within a double-membrane system composed of the outer and inner mitochondrial membrane. MtDNA contains 37 genes, of which 13 protein components are involved in OXPHOS [19, 20]. It has been reported that mtDNA mutations, including an overall reduction and increased variability of contents in PCa cells, would deteriorate OXPHOS, thus increasing the production of ROS [21–23]. Previous research reported that approximately 11–12 percentages of PCa patients manifested mutational cytochrome oxidase subunit I (COI) with significant functions [24]. Additionally, high levels of mitochondrial complex I-encoding genes mutation of PCa decrease 70% NADH-pathway capacity and increase ROS levels, particularly in high-grade PCa [25]. Remarkably, prostate tumors implanted subcutaneously with the pathogenic mtDNA ATP6 T8993G mutation of the PC3 cells were seven times larger than the wild-type (T8993T) cybrids; the mutant tumors also generated significantly more ROS [26]. Furthermore, ROS can attack polyunsaturated fatty acids in membranes to trigger mtDNA leakage [27]. Lack of histone protein protection and damage-repair mechanisms, the exposed mtDNA is prone to mutations induced by ROS, which is called ROS-induced ROS-release and causes a vicious cycle [28].

### 2.2. NADHP Oxidases (NOXs)

NOX is a complex membrane protein consisting of the catalytic subunits gp91phox, p22phox, regulatory subunits p40phox, p47phox, p67phox, and the small GTPase Rac [29, 30]. The NOX family comprises seven isoforms: NOX 1–5 and dual oxidases (DUOX) 1 and 2 [31]. NOXs catalyze the transfer of electron across biological membranes via electron donor NADPH and are responsible for ROS generation, which includes both superoxide and hydrogen peroxide [32, 33]. NOX1, NOX2, NOX4, and NOX5 expressions are increased explicitly in a high percentage of PCa cells compared to benign cell lines, consequently contributing to PCa survival and progression via ROS-regulated signaling cascades [34, 35]. ROS produced by NOX4 mediate the antiapoptotic effect of growth factors [36]. Although having similar structures, the NOXs are activated by specific mechanisms and regulatory subunits, respectively [37]. Especially, as NOX2 and NOX4 mRNAs are androgen-dependently regulated, radiotherapy has shown a significant benefit in metastasis-free survival when used in combination with ADT at early stages [38]. These findings collectively suggest that exploring specific antisense targeting of NOX enzymes or NOX enzyme inhibitors may represent a valuable strategy for PCa treatment by modulating the NOX-dependent intracellular redox status.

### 2.3. p66Shc

p66shc, a prooxidant isoform of the ShcA adaptor protein family, has the same modular structure of p52Shc/p46Shc (SH2-CH1-PTB) and an additional N-terminal CH2 domain containing a particular phosphorylated serine residue at position 36 (Ser36) [39, 40]. Oxidative stress induces ser36 phosphorylation to trigger p66Shc activation, which, in turn, promotes electron transfer from cytochrome c to oxygen, thereby increasing the generation of hydrogen peroxide [41, 42]. p66shc also leads to ROS generation by increasing NOXs levels or impairing intracellular antioxidant levels indirectly through inhibiting the activities of FOXO transcription factors [43]. Clinical prostate tumors show higher levels of p66Shc, relative to adjacent noncancerous specimens, which implies its vital tumorigenic role [44]. In CRPC cells, elevated p66Shc increases oxidant species production to maintain cell proliferation under androgen-deprived conditions [45]. Besides, p66Shc plays a crucial role in the migration of CRPC cells via ROS-induced activation of Rac1 [45, 46]. However, many other studies reveal that p66Shc is also regarded as an apoptotic mediator independent of the adapter function [47]. Overexpression of p66shc mediates excessive ROS generation and Akt/PKB dephosphorylation, ultimately inducing PCa cell death [48].

## 3. Cellular Detoxification from ROS of PCa

Enzymatic or nonenzymatic antioxidants involved in scavenging of different types of ROS play crucial roles in protecting tissues and cells from free radical-mediated oxidative damage [7]. Kelch-like ECH-associated protein 1 (Keap1)–Nrf2/antioxidant responsive element (ARE) acts as an essential modulator initiating antioxidant defenses and contributes to the progression of several tumors [49]. As a specific negative regulator, Keap1 binds to Nrf2 in the cytoplasm, thus inducing Nrf2 ubiquitination and subsequent degradation by the proteasome. While oxidative stress dissociates the Nrf2–Keap1 complex, the transcription Nrf2 transfers into the nucleus and combines with ARE in the promoter regions of the downstream genes to activate the transcriptional expression of antioxidant enzymes [50, 51]. The targets of Nrf2 refer to superoxide dismutase (SOD), catalase (CAT), glutathione peroxidase (GSH-Px), and heme oxygenase-1 (HO-1), which constitute the primary endogenous antioxidant defense system located in the mitochondria and cytoplasm [52]. SOD and CAT are generally functioned against elevated superoxide anion and hydrogen peroxide, respectively [53]. Nonenzymatic scavengers mainly include thioredoxin (Trx), glutathione (GSH), as well as low-molecular-weight antioxidants like cytochrome c and coenzyme Q. The process that GSH is oxidized to GSH disulfide (GSSG) through the interaction with GSH S-transferase directly or via a reaction catalyzed by GSH-Px could alleviate oxidative damage through decreasing disulfide bonds of cytoplasmic proteins to cysteines [54]. Excessive ROS can induce an oxidized Trx form, which is subsequently converted to a functionally reductive form by thioredoxin reductase (TrxR) to maintain redox homeostasis in cells [55] (Figure 1). Despite lower antioxidant capacity as compared with normal cells, PCa cells adaptively synthesize more antioxidants like HO-1, Nrf2, and GPXs to cope with the continued ROS production. A wealth of studies have suggested that under the dynamic nonequilibrium of ROS, elevated antioxidant genes facilitate the maintenance of protumorigenic signaling and protect against oxidative-dependent death within tumor cells [56]. There is a 45% failure of PCa patients after high-dose radiotherapy against localized diseases, which may be partially due to elevated basic Nrf2 gene expression essential to resist hazardous environmental insults [57]. Overexpression of antioxidant gene KLF4 restores the redox balance of PCa cells and reduces ROS-dependent cell death induced by chemotherapy drugs, such as high concentrations of H2O2 and paraquat [56, 58]. The silence of the KMTD2 gene could weaken the combination of antioxidant genes with FOXO3 DNA to downregulate the expressions of antioxidants, thereby enhancing the chemosensitivity of PCa cells [59]. MiR-17-3p inhibits expressions of mitochondrial antioxidant enzymes to reduce the radioresistant capacity of PCa cells [60]. In conclusion, we could pay attention to the significant role of antioxidant genes in the development of resistance to oxidative stress in PCa and develop new efficient drugs targeting antioxidants.

## 4. Roles of ROS Molecules in PCa

A moderate level of ROS guaranteed by redox balance is essential for physiological activities via the activation or inactivation of metabolic enzymes, as well as the regulation of calcium in mammalian cells [61]. Once the redox status deviates to oxidation, increased ROS can cause oxidative damage and regulate signaling pathways, further affecting several cancer hallmarks such as survival, proliferation, angiogenesis, invasion, and metastasis in a concentration-dependent manner [35]. In a study performed on PCa cell lines, the proliferative activity of LNCap cells exposed to low concentrations of H2O2 increases. Still, it returns to the pretreatment level after continued exposure to the antioxidant HDL that can counteract the elevated ROS induced by H2O2 [62]. Furthermore, according to the redox imbalance of tumor cells, we can filter several indicators including increased 8-hydroxydeoxyguanosine or F2-isoprostane in urine and decreased levels of the antioxidant-tocopherol or increased peroxide levels in serum as diagnosis and prognosis markers in PCa [63].

The mechanisms of ROS on the biological manifestation of PCa have been vividly discussed in the latter sections. An excessive or extremely deficient level of ROS increases the chances of cell death or inhibits cell growth through mediating ROS-dependent signaling cascades, which represents a novel anticancer therapeutic strategy based on ROS regulation.

### 4.1. ROS and Prostate Carcinogenesis

Tumorigenesis is associated with genotype changes and progressive abnormalities of phenotype. In general, a higher level of ROS in PCa causes oxidative damage of crucial cellular constituents (proteins, lipids, DNA, and RNA), further inducing gene mutation and abnormal activation of cellular signaling pathways, eventually contributing to the early events involving tumorigenesis and tumor progression.

ROS lead to DNA damage through mediating single or double-strand breakage as well as pyrimidine and purine lesions [64]. The accumulation of DNA damage via incomplete repair or misrepair can disrupt genome stability and trigger consequently transformation, especially if combined with a deficient apoptotic pathway [65]. Furthermore, numerous reports have described that ROS, as a direct DNA mutagen, activate several oncogenes (receptor tyrosine kinases, Src, and Ras) and inactivate several tumor suppressor genes (PTEN, p53, and TSC2), thus contributing to malignant cellular transformation and the activation of stress-responsive survival pathways [66, 67]. Profound cellular oxidative stress induces lipid peroxidation, promoting the generation of 4-hydroxy-2-nonenal and 1, N6-ethenodeoxyadenosine, which subsequently facilitated mutations of the p53 [68, 69]. Conversely, the active K-ras and deficient p53 further accelerate the ROS accumulation through leading to mitochondrial dysfunction or induction of NOX family proteins, which is necessary for their tumorigenicity [70–73]. Nox5-derived ROS mediate the proliferation and survival of PCa cells through enhancing PKC*ζ* expression and inducing phosphorylation of JNK1/3 [74]. Moreover, several proteins translationally lose regulatory functions due to ROS-dependent modifications of cysteine residues, such as disulfide formation, S-nitrosylation, and reversible glutathionylation [75]. PTEN, as a representative tumor suppressor, is dysregulated in PCa, and PTEN deletion is already characterized by a poor prognosis [76]. Mechanically, ROS can induce the formation of a disulfide bond between the active site cysteine (C71) and another adjacent cysteine (C124) to suppress PTEN activity, thus activating constitutively AKT signaling and further enhancing aberrant growth of the PCa [77]. A previous experiment observed ROS increased CXCR4-mediated metastasis via the inactivation of PTEN in PCa cells [78].

Epigenetics is regarded as mitotically heritable changes in the expression of genes that maintain the intrinsic DNA sequences. Previous studies suggested ROS may be involved in epigenetic instability/cascade to initiate carcinogenesis, which was a near-universal feature of human cancers [79, 80]. ROS increase the expression of DNA methyltransferases (DNMT) enzymes that either catalyze the transfer of a methyl group to DNA or speed up the reaction of DNA with the positive-charged intermediate S-adenosyl-L-methionine through deprotonating the cytosine molecule at the C-5 position in the process of DNA methylation [81, 82]. Recent evidence shows that overexpression of DNMT plays critical roles in progression, metastases, and therapy resistance of PCa, particularly in advanced stage [83, 84]. ROS can evoke the repression of CDH1 to enhance the epithelial-mesenchymal transition (EMT) process through methyl modification of chromatin [85]. Furthermore, ROS accelerate progression to a malignant phenotype through mediating histone modification that is mainly dependent on histone acetyltransferase (HAT) and histone deacetylase (HDAC). Histone H3 acetylation regulated by ROS promotes the EMT process [86]. As enhancer activity markers, histones acetylation (H3K27ac, H3K9ac) may modulate antioxidative gene transcription by adjusting the spatial structure of chromatin [87]. Besides, it has been reported that decreased overall histone acetylation or elevated nuclear levels of acetylated histone 2A.Z were closely associated with poorer outcomes of PCa [88–90].

ROS function as redox messengers at modest levels to mediate PCa progression via regulations of various signaling molecules. Many transcription factors that include HIF-1, NF-*κ*B, and AP-1 are redox-sensitive, and thiol oxidation of these proteins can alert their DNA-binding activity to have an indirect effect on DNA [91]. After elevated intracellular ROS levels, stabilization of HIF-1*α* plays a vital role in cell transformation [36]. ROS can activate NF-*κ*B/IL-6/IL-8/pSTAT3 pathway to enhance the proliferation and metastasis of PCa cells [92–94]. Also, AP-1 has been described to regulate the initiation and recurrence of prostate cancer via activating constituent downstream genes like c-Jun and c-Fos [95].

Additionally, the raised levels of mitochondrial ROS induce abnormal activation of mitogen-activated protein kinase (MAPK)/extracellular-signal-related kinase (ERK) [96–98] for survival and the increased resistance to apoptosis [99]. As mentioned above, the dismantlement of the Nrf2-Keap1 complex is due to the oxidized cysteine residues of Keap1 induced by ROS. Besides the effect of ROS detoxification, Nrf2 activation increases cell viability and improves the invasive and migratory abilities of PCa cells via EMT [100]. In conclusion, inhibitors of ROS generation in PCa cells could effectively suppress genetic instability and initiation of redox signaling cascades, resulting in fewer metabolic adaptations and less proliferation and survival.

### 4.2. ROS and Androgen Receptor(AR)

AR is a nuclear receptor transcription factor with the three-dimensional crystal structure containing the ligand-binding domain (LBD) and DNA binding domain (DBD). It is essential to aggressiveness and progression of PCa [101]. Androgens activate AR signaling by binding to AR to drive the growth as well as metastasis and simultaneously suppress apoptosis of PCa cells [102–104]. Previous studies have shown that ROS production or oxidative stress-associated markers are required for androgen stimulation in AR-positive cells. ROS have been proposed to stimulate the AR nuclear translocation and AR-mediated transcriptional activity via inducing PTEN loss [105]. There is close proximity as well as the overlap between AR response elements and binding sites for NF-*κ*B, so ROS-mediated activated NF-*κ*B may bind directly to the AR promoter to alter AR DNA binding activity and its downstream gene transcription [106].

The commonly targeted genes of AR signaling contain prostate-specific antigen (PSA), B-cell lymphoma-extra large (Bcl-xL), and NK3 homeobox 1 (NKX3.1), which are highly expressed in metastatic PCa and CRPC [107]. The increased levels of PSA in serum are considered as a sensitive marker for the development and progression of PCa [108]. PSA releases insulin growth factor-1 (IGF-1), thus catalyzing IGFBP-3 to promote the proliferation of PCa [109]. ATD remains a routinely adopted therapy for locally advanced and metastatic prostate cancer through inhibiting the androgen biosynthesis or preventing androgen from binding to AR. However, after a period of treatment, the majority of patients eventually progress into CRPC which is primarily driven by the aberrant AR activities including AR gene amplification, mutations on AR gene ligand-binding domain, and elevated AR coactivators as well as AR splice variants [110–112].

Recent studies indicate that androgen effects might not be equal to the AR effects. Besides, androgen-independent (AI) cells have a higher level of oxidant species than androgen-sensitive (AS) cells, which suggest that ROS can cause deregulations of the AR axis pathway [113]. It is reported that AI PCa cells exhibited higher p66Shc protein levels that activate NOX complexes and stimulate mitochondrial superoxide production for intracellular ROS generation to a high degree [45]. Additionally, there is a lower glutathione (GSH) content and GSH/glutathione disulfide ratio in PC-3 cells that serve as a representative of AI PCa cells [114]. In comparison to the C4-2B/LNCaP cells, PC-3 cells show a significant increase in Trx1 protein levels; however, the decrease of total Trx activities and higher oxidation of Trx1 resulting from reduced TrxR1 or increased TXINP, also correlated with higher levels of ROS in PC-3 cells [115]. Inversely, the upregulated ROS levels accelerate the proliferation and metastasis of PC-3 cells via mediating the specific absence of the P53 gene and PTEN gene, as well as the constitutive activation of PI3K/AKT signaling [116–118]. ROS positively modulate AR expression or possibly AR mRNA stabilization [112]. ROS not only upregulate TXNDC9 expression for MDM2 degradation but also enhance PRDX1-mediated AR protein stabilization and subsequent AR signaling transactivation [119]. Antioxidant Trx1 inhibition also elevates ROS-dependent AR levels of CRPC when combined with ADT [120]. Overexpression of Nrf2 can suppress AR expression and function in PCa cells via decreasing ROS levels [121]. Under the castrated levels of androgens, hypoxia enhances the transcriptional activity of AR through ROS-mediated HIF-1*α* [122]. Alternately, due to mutations of the ligand-binding domain (LBD) partially induced by ROS, abnormal activation of AR signaling also occurs in response to growth factors, cytokines, and kinases, which disengages tumors from hormone-dependent environments. Targeting the AR for direct degradation may lead to better efficacy to further suppress the PCa progression. Enzalutamide, an FDA-approved targeted AR inhibitor, is commonly prescribed to prolong overall and progression-free survival in patients [123]. However, some limitations by the resistance of such intrinsic drugs eventually cause the failure of therapy. More pieces of evidence demonstrate that the emergence of variant types of AR is associated with the progression of CRPC, and reversing the phenomenon could improve the prognosis of PCa [124]. ROS has been shown to induce splice variants of AR and augment AR-Vs-expressions via mediating NF-*κ*B activation in PCa cells [106]. Additionally, ROS could have a direct effect on the expression of several splicing factors like heteronuclear ribonucleoproteins (hnRNPs) that play critical roles in AR expression and production of variants in PCa [125]. Despite lacking the ligand-binding domain, the most significant AR-V7 remains constitutively active under the castrated levels of androgens. It stimulates the transcriptional activation of AR target genes as it still retains the transactivating N-terminal domain (NTD) [126]. Conversely, AR expression is vital for redox homeostasis [127]. Activated AR pathway facilitates ROS production most strongly in an environment deficient of androgen. AR signal mediates malignant biological behaviors of CPRC at least in part by stabilizing the posttranslation of p66shc and increasing p66Shc protein levels [128].

Contradictorily, some evidence reveals that extremely high levels of ROS could negatively regulate the translational levels of AR. Isoselenocyanate-4 (ISC-4) inhibited LNCaP cell growth and survival via ROS-mediated suppression of AR and PSA abundances without initially decreasing their steady-state mRNA level [129]. ABT263 drug could increase ubiquitin/proteasome-dependent degradation of AR and AR-v7 proteins through the ROS/USP26 axis, enhancing CRPC cell sensitivity to Enzalutamide [130]. Besides, acute exposure (2 h) to CDDO-Me increased ROS levels to suppresses AR and its splice-variant AR-V7 at both the transcriptional and translational levels [131].

There seems to be a regulatory loop between AR and intracellular ROS, which suggests that AR activity is regulated by ROS and AR signaling functions via mediating ROS generation. Further exploration of specific crosstalk between ROS and AR has been shown broad prospects of treatments for PCa.

### 4.3. ROS and Tumor Microenvironment (TME)

The TME is extraordinarily complex and dynamically variable [132]. Compared to adjacent healthy tissue, tumors are known to have a highly oxidative microenvironment, which may play a crucial step in the interactions between tumor cells and the surrounding stromal cells. TME is mainly divided into two aspects: nonimmune microenvironment dominated by fibroblasts and immune microenvironment based on immune cells. It is generally accepted that PCa cells acquire a symbiotic relationship with TME. The reciprocal crosstalk between them occurs via various intercellular communications such as direct cell-to-cell contact, migration of extracellular vesicles (EVs), and chemokines/cytokines secretion partially induced by ROS, jointly leading to tumorigenesis and progression [133, 134]. Lysophosphatidic acid LPA of TME binding to LPA1–3 receptors of PCa cells promotes calreticulin (CRT)/vegf-c expression to induce lymphangiogenesis and lymphatic metastasis through ROS-mediated phosphorylation of eukaryotic translation initiation factor 2*α* (eIF2*α*) [135]. ADT induces the migration of mesenchymal stem cells (MSCs) into tumor tissue via the ROS/NF-*κ*B/IL-1*β* pathway of PCa cells. MSCs, in turn, increase the stemness of PCa cells via secreting chemokine ligand 5 under the AD condition [136].

As a significant component of tumor stroma, cancer-associated fibroblasts (CAFs) promote the proliferation and metastasis of PCa cells through the TGF-*β* pathway [137]. CAFs have been revealed to enhance the numbers of PCa stem cells and be involved in the PCa angiogenesis and chemoresistance [138]. Moreover, CAFs increase glutathione levels of PCa cells to counteract drug-induced oxidative death [139]. Emerging evidence suggests TGF*β*1-mediated CAFs activation is associated mainly with Nox4-derived ROS signaling [140]. Redox-dependent CAFs activation has the immunosuppressive function via phosphorylation of JNK [140–143]. CAFs broadly suppressed immune response by explicitly excluding CD8+ T cells from tumors through upregulating NOX4 levels [144]. Similarly, NOX4-mediated ROS play a key role in CAFs-induced functional cell reprogramming from monocytes into immunoinhibitory MDSCs that inhibit T-cell proliferation and impair T-cell function [145].

As a prominent component in infiltrating immune cells, tumor-associated macrophage (TAM) accounts for up to 70% of prostate tumor immune subsets [146]. Macrophages are well known due to their heterogeneity and plasticity, which generally polarize towards two extremes, the tumor-suppressing M1 phenotype or tumor-promoting M2 phenotype. The recruitment and functional evolution of macrophages in TME can be modulated by various cytokines, tissue factors, and conditions [147]. CCL2-secreting CAF facilitates the recruitment of TAM from systemic sites to the microenvironment of PCa [148]. ADT induces ROS-dependent expression of colony-stimulating factor 1 (CSF1) that leads to a significant enhancement of TAM infiltration and skews them towards the M2 phenotype in PCa [149]. On the other hand, the soluble mediators released by PCa cells could aid in polarization to the M2 phenotype, such as IL-6 [150, 151]. Hypoxia enhances the Warburg effect of PCa cells via HIF-1 expression, thus inducing secretion of exosomes rich in lactate, which could promote TAM towards the M2 phenotype [152]. Several studies specifically implicate that high percentages of activated M2 phenotype in the TME are a hallmark of cancer, and usually predict poor clinical prognosis in PCa patients. As such, PCa patients with elevated M2-TAMs infiltration have shown an increase in the probabilities of dying [153]. A wealth of studies have revealed immune cells release profound cytokine to stimulate NOX-mediated ROS production within tumor cells, which alters DNA integrity and enhances the angiogenic process [154]. M2-phenotype-secreted CCL5 results in PCSCs self-renewal and PCa cell metastasis via activating *β*-catenin/STAT3 signaling [155].

Indeed, the M1 phenotype enhances phagocytosis by ROS-mediating NF-*κ*B activation and tolerates a broader range of ROS levels [156]. However, despite having lower ROS levels than the M1 macrophages, M2 macrophages still require moderate ROS for polarization and become more vulnerable to alterations in cellular redox status. Luput et al. reported the significant role of NADHP oxidase in the modulation of the protumor actions of M2-macrophages [157]. The ROS generation in M2 macrophages is required for the synthesis of MM2 and MMP9, which is followed by the metastasis of PCa cells. Additionally, M2 macrophages exhibit elevated expressions of some crucial antioxidants [158]. Nrf2 activation of M2 macrophages increases vascular endothelial growth factor (VEGF) expression and contributes to the EMT process of tumor cells [159]. Given the key redox differences, ROS scavengers can decrease ROS levels to attenuate polarization of the M2 but not the M1 macrophages, such as MnTE and the pan-Nox inhibitor, diphenyleneiodonium (DPI) [158].

As signal molecules, ROS may decrease PCa cell immunogenicity by bypassing the surveillance of immune cells. In PCa cells, ROS-induced PTEN loss increases IDO1 protein expression and FoxP3+ Treg density of TME, thereby triggering an immunosuppressive state and promoting tumor growth and invasion [160, 161]. High CD8+ T cells infiltration correlates with a good prognosis due to their cytotoxic functions in many solid tumors [162, 163]. However, vast stromal CD8+ T cells are associates with poor prognosis in radical prostatectomy specimens and shorter time until BCR in PCa patients [164]. These findings indicate that CD8+ T cells in the microenvironment of PCa may be senescent, dysfunctional, or suppressed. Mechanically, nonfunctional CD8+ T cells upregulate their negative coinhibitory markers or downregulate the positive costimulatory molecules, thereby resulting in the suppression of antitumor immune responses [165]. Previous preclinical studies have reported that overexpression of lymphocyte activation gene-3 (LAG-3) as the coinhibitory molecules on CD8+ T cells can regulate T-cell tolerance to tumor antigens [166]. In particular, the PD-1/PD-L1 axis acts as a crucial regulator of immune checkpoints to suppress the adaptive immune system. The PD-1 is mainly expressed on T cells, and its ligand PD-L1 is commonly expressed on tumor cells. Once PD-1 binds to PD-L1, PCa cells block the active cytotoxic function of T lymphocytes through immune evasion [10]. Emerging evidence demonstrates that ROS have a significant influence on the expression of PD-1 and PD-L1. An enhanced generation of ROS usually promotes PD-L1 expression on the surface of tumor cells as well as PD-1 expression on T cells via multiple signaling factors such as HIF-1, JAK/STAT3, and NF-*κ*B [167]. Inversely, ROS scavenging directly represses their expressions in general. Furthermore, a potent ROS scavenger also selectively inhibits M2 macrophage polarization, indirectly limiting or decreasing the expression of PD-L1 [167]. It is noteworthy to investigate the specific mechanism of the effect of ROS on TAM differentiation and regulation of the PD-(L)1 immune checkpoint.

### 4.4. ROS and Cytoprotective Autophagy

Autophagy is a “self-feeding” phenomenon that allows lysosome to degrade damaged, senescent, or nonfunctional proteins and organelles. It is an evolutionarily conserved biological process in eukaryotic cells and plays a vital role in maintaining cell homeostasis and renewal [168, 169]. In healthy cells, autophagy proceeds at a basic level to prevent tumor initiation by inhibiting inflammation and chronic tissue damage and maintaining genome integrity [170]. Nassour demonstrated that both insufficient and absent autophagy was necessary for tumorigenesis [171, 172]. Monoallelic loss of the essential autophagy gene BECN1, MAPLC3, and ATG5 has been frequently found in PCa. In part, deletions of BECN1and ATG5 are a driver of prostate tumorigenesis via disordered degradation of damaged mitochondrial and ROS-mediated DNA damage [37]. In contrast, autophagy has recently emerged as a critical regulator of multiple processes of cancers and is usually correlated with the development and progression of tumors. In cancerous cells where malignant transformation has been completed, elevated autophagy can provide anabolic energy and raw materials through recycling components of nonfunctional organelles to mediate the growth of tumor cells [173, 174]. Tumor cells can evade apoptosis through autophagy regulation, thus increasing drug resistance and enhancing tumor cell viability [175]. Although many cancers, such as prostate cancer, exhibit elevated autophagy levels, the regulatory mechanisms of this process are still not clear.

A recent report reveals 35% of PCa patients with a high Gleason score (GS) show an increase in the vital autophagy proteins (p62). It has been identified that genetic alterations and androgen are responsible for autophagy activation in PCa. The lysine demethylase KDM4B significantly increases the LC3 puncta and the protein levels of LC-3II by activating Wnt/*β*-catenin signaling, which indicates that upregulated KDM4B facilitates autophagy activation. Importantly, specific autophagy inhibitor (3-MA) partially attenuates KDM4B-induced CRPC cell proliferation [176]. Furthermore, overexpression of NPRL2 promotes docetaxel chemoresistance of CRPC cells by regulating autophagy via mTOR signaling [170]. Also, androgen induces autophagy and autophagic flux of PCa cells through the AR pathway to promote cell proliferation [177, 178]. Indeed, the mRNA and protein levels of 4 core autophagy genes: ULK1, ULK2, ATG4B, and ATG4D are upregulated by androgen and correlate with poor prognosis of PCa [177].

One of the downstream processes affected by redox imbalance is autophagy. Currently, some significant modes of ROS-regulated autophagy have been revealed. The oxidation and inactivation of Atg4A by ROS leads to the conjugation of LC3 to phosphatidylethanolamine, inducing autophagy activation [179]. Additionally, ROS directly upregulate expression of BNIP3 via activating HIF-1, thus inhibiting mTOR activity that is negatively associated with autophagy activation [180]. Inhibition of mTOR is also generated by activated TSC2 due to ROS-mediated the oxidation of ataxia telangiectasia mutated (ATM) [181]. In contrast, autophagy is a self-defense mechanism by which PCa cells withstand excessive oxidative stress. Especially when there exists a high level of p62 in PCa cells, autophagy can cause the degradation of Keap1 depending on the direct physical interaction between Keap1 and p62, thus limiting ROS amplification through Nrf2/ARE axis [182, 183].

Recent shreds of evidence demonstrate that autophagy activation is generally accompanied with ROS that function as crucial molecules in the crosstalk between autophagy and apoptosis [184]. Mechanically, excessive ROS generate an autophagy-dependent cytoprotective response through inducing activations of multiple signalings, such as AMPK/ERK and NF-*κ*B, which attenuates original ROS-mediated apoptosis [185]. Besides, ROS induce the phosphorylation of beclin1 and Bcl-2 through abolishing the interaction between them, thus accelerating the activation of autophagy and apoptosis [186].

According to reports in the literature, various anticancer drugs, such as lasalocid and adriamycin, have been confirmed to activate the ROS-dependent autophagy, which has negative impacts on their proapoptotic effects. Resulting in cytotoxic apoptosis of PCa cells, lasalocid simultaneously induces ROS-dependent cytoprotective autophagy. Thus, autophagy inhibitor (3-MA) enhances lasalocid-induced apoptosis, which might result from elevated ROS production [184]. Similarly, the combination of adriamycin with the late phase autophagy inhibitor (CQ) resulted in more pronounced tumor suppression of PCa cells [187]. These results indicate that ROS-mediated autophagy acts as a protector for PCa cell survival. In this context, it could be assumed that the addition of agents that inhibit ROS-reactive cytoprotective autophagy enhances the proapoptotic effect of various cancer therapies [188].

Indeed, autophagy, as a “double-edged” sword, plays a complex and paradoxical role depending on different stages of cancer development and cell type [188]. The cytotoxic autophagy triggers cell death (named autophagic cell death), which will be discussed in detail below.

### 4.5. ROS and Cell Death

Due to a lower capacity of the antioxidant system, tumor cells are more sensitive to fluctuations in ROS levels than healthy cells. This accumulation of cellular ROS upon overwhelming amounts may induce secondary oxidative damage and lead to various types of PCa cell death including apoptosis, autophagic cell death, necrosis, and ferroptosis. Emerging evidence indicates that several anticancer agents require upregulation of ROS levels to mediate tumor cell death. Therefore, increasing intracellular levels of ROS over a threshold could be a novel therapeutic strategy.

Apoptosis, also known as type I genetically programmed cell death, is a normal biological process described by stereotypical morphological alterations involving nuclear fragmentation and condensation, membrane blebbing, and apoptotic body formation [189, 190]. Two significant apoptosis pathways have been reported: the mitochondria-mediated pathway and death receptor-mediated pathway, which depend on the caspase activation [191]. A wealth of studies highlight ROS serve as a significant role in chemotherapy and radiotherapy against various cancers. It has been proved that high levels of ROS above a toxic threshold cause mitochondrial dysfunction and activate death receptors [192, 193]. Mitochondria are both the primary source of ROS generation and the pivot of intrinsic apoptosis regulation. ROS can trigger the opening of permeability transition pore on the mitochondrial membrane by regulating the Bcl-2 family, thus leading to increased mitochondrial membrane potential (MMP) loss, which is thought to be an early event and a possible cause of programmed cell death [194, 195]. In addition to blocking cell cycle at G1 phase, which is partly associated with ROS-mediated cell injury, oleanolic acid methyl ester (OAME) also induces ROS-dependent MMP loss, the release of cytochrome c, and activation of caspase 7/3. These caspases mediate the execution phase of apoptosis with a cascade of proteolytic activity. It indicates that OAME triggers ROS-mediated apoptosis of PCa cells through targeting the mitochondrial pathway [196, 197]. Due to the loss of cytochrome c from the mitochondria, profound cytochrome c forms a complex with apoptotic protein-activating factor 1 (Apaf-1) to activate caspase cascades and further increases production of ROS following disrupting the mitochondrial ETC [198]. The extrinsic pathway is activated upon binding of proapoptotic ligands to corresponding death receptors including Fas, TNF receptor 1 (TNFR1), TNF-related apoptosis-inducing ligand receptor 1 (TRAIL-R1), and TRAIL receptor 2 (TRAIL-R2) [199]. ROS may induce the DNA damage-dependent ATM and ATR activation of PCa cells, upregulating the expression of DR5 (TRAIL-R2) and Fas (CD95) proteins on the membrane, thus resulting in caspase 8 activation/PARP cleave and subsequently triggering apoptotic pathway. Furthermore, ROS mediate TRAIL/FasL signaling between NK cells and tumor cells to enhance the lethality of NK cells [119].

Additionally, a galaxy of research findings have demonstrated that ROS act as upstream signaling molecules to hinder accurate protein folding processes and disturb endoplasmic reticulum (ER) homeostasis, which can be called as ER stress. Severe ER stress has the ability to initiate another atypical intrinsic apoptosis response [200]. Induction of ER stress activation and PCa cell apoptosis by both Chelerythrine (CHE) and Isoalantolactone (IALT) is dependent on ROS generation [193, 201]. Mechanically, IALT increases the levels of ROS-dependent p-eIF2*α* and ATF4 in the PC-3 and DU145 cells, thus stimulating expression of the transcription factor CHOP that inhibits the expression of Bcl-2 and is strictly responsible for the initiation of the cell apoptosis cascade [201–203]. Mitochondrial outer membrane permeabilization (MOMP) also results in elevated cytoplasmic proapoptotic molecules containing apoptosis-inducing factor (AIF) and endonuclease G (Endo G) in response to organelle damage induced by ROS, and these molecules function in a caspase-independent manner [204]. As such, Auriculasin-induced ROS initiate apoptosis of PCa cells through the elevated release of AIF and Endo G via the depolarization of the mitochondrial membrane [204].

As mentioned above, autophagy can also function as a tumor suppressor mechanism in response to various stressors like oxidative stress (179). Autophagy-associated cell death, especially autophagic cell death, is called type II programmed cell death and partly results from mitochondria dysfunction [205, 206]. Once ROS levels surpass the cellular antioxidant capacity, autophagy may fail to remove the excess ROS that persistently damage mitochondria, resulting in autophagy-associated cell death. Additionally, continuous or excessive induction of autophagy serves as a “pro-death” signal, leading to inordinate cell degradation and self-digestion of vital cellular components via accumulation of autophagic vacuole, eventually resulting in autophagic cell death in a caspase-independent pathway [207, 208]. An arsenic compound KML001 induced ROS-dependently upregulation of autophagic specific protein LC3, which is followed by an increase in cell death (autophagic cell death) [209]. Furthermore, the induction of autophagic cell death by small molecules can enhance the antitumor activity of radiation therapy or chemotherapy.

As nonprogrammed cell death, necroptosis is initially described as a passive mechanism of cell demise. It is characterized by the morphological traits containing rounding of the cell, organelle swelling, plasma membrane rupture, and leakage of nuclear constituents with the inflammatory surrounding [210, 211]. Cancer cells preferentially depend on glycolysis (Warburg effect) for ATP production in hypoxia conditions, which endows selective advantage in the presence of diminished nutrition but results in tumor cells more sensitive to glycolysis inhibition [212]. The glucose analog 2-deoxy-d-glucose (2DG), an inhibitor of glycolysis and glucose transport, can reduce intracellular ATP levels and cause elevated ROS generation, finally culminating in necrotic cell death [213]. A single agent 2DG can induce cytotoxic effects on PCa cells [214]. Furthermore, various evidences have proved that the key enzymes in glycolysis, such as HK2, PFK, and PK, play vital roles in the survival of PCa cells [215]. Selenite induces necrotic cell death of PCa cells through triggering ATP depletion via inhibiting PFK activity, whereas N-Acetyl-cysteine (NAC) can rescue selenite-induced ATP depletion and PFK activity, which indicates that ROS are involved in necroptosis through inhibiting PFK activity directly or indirectly [215].

Further researches have revealed that necrosis is a regulated process critically dependent on a complex consisting of RIP1, RIP3, and MLKL [210, 216]. Necroptosis is usually accompanied by an intense burst of ROS production. However, it is not the direct executioner of necroptosis [217]. Recruitment and activation of RIP3 dependent on RIP1 phosphorylation can lead to MLKL phosphorylation through ROS generation by the activation of the pyruvate dehydrogenase complex [216]. ROS-dependent MLKL activation triggers its oligomerization and membrane translocation to stimulate the formation of pores and the influx of ions (mainly calcium) on the membrane, eventually resulting in the rupture of cell membranes and cell death [218, 219].

Ferroptosis is characterized by the accumulation of lipid hydroperoxides (LOOH) and high expression of HO-1 in an iron-dependent manner [220]. While accompanying with augmented lipid peroxidation and glutathione depletion, excessive antioxidant HO-1 may behave in prooxidant compounds following a direct reaction with ROS in the conditions of transition of metal ions such as copper and iron, eventually leading to cell death through a process called as ferroptosis [99]. ALZ003 potently triggered the ferroptosis of PCa cells by impairing AR-regulated GPX4 that is a GSH-dependent enzyme required for the elimination of lipid [221].

### 4.6. Challenges and Opportunities Related to the Chemoprevention of PCa by the Antioxidants

Epidemiological evidence strongly suggested that a lower risk of cancer was associated with higher consumption of vegetables and fruits [222]. Therefore, the researches of naturally available pharmaceutical agents against PCa are of particular interest. Several clinical trials pointed out the properties of the popular antioxidants, such as some minerals (selenium), vitamins, and polyphenols, and showed their encouraging results against PCa prevention (Table 1). However, some contradictory data questioned the clinical effects of antioxidants on human health. The Selenium and Vitamin E Cancer Prevention Trial (SELECT), a large intervention study, revealed that the supplement of selenium + vitamin E had no effect on reducing PCa risk. Surprisingly, single vitamin E supplementation increased the risk of PCa [223, 224]. In a separate study, higher baseline selenium was associated with a higher risk of increased PSA velocity in nonmetastatic PCa [225]. Grant has observed a positive relationship between vitamin D intake and PCa [226]. The different results may be due to improper dosage, formulation, intervention periods, and patient populations. Anyhow, there is a large quantity of challenges and opportunities in the antioxidative treatment models for PCa prevention. The possible application of any discovery seems staggering in the field of public health. Further clinical studies are warranted to carry out a large-scale cohort study in multiple regions and control several potential confounders in the analysis. Eventually, we select an optimal combinatorial approach of antioxidants against different individuals to reduce the risk of morbidity and mortality of PCa.

## 5. Conclusions

**Figure 2:**
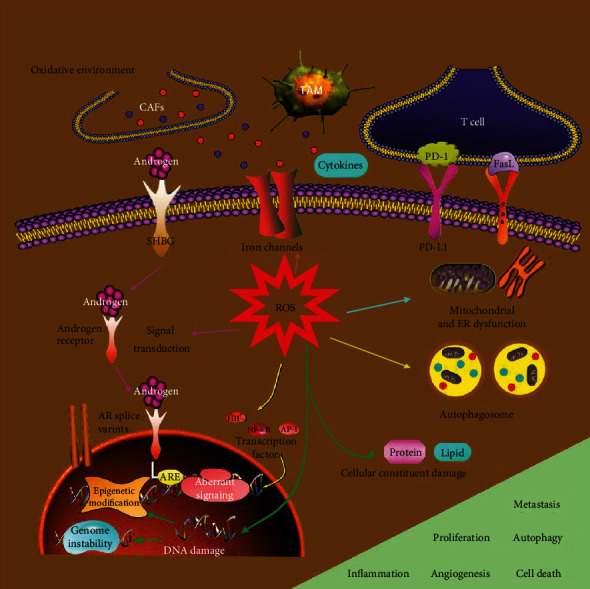
The downstream cellular effects of ROS. ROS are believed to be implicated in the initiation and progression of PCa. Cellular excessive ROS result in constituent damages in DNA, proteins, and lipids beyond repair, thus leading to gene instability and epigenetic modification. Furthermore, ROS mediate aberrant signaling pathways through changes in the activity of membrane receptors, ligands, ion channels, and transcription. One of the downstream processes affected by ROS is autophagy. Especially, ROS are involved in androgen signaling transduction and regulate the expression of AR splice variants. Additionally, the oxidative microenvironment of PCa consists of a group of various nonmalignant cells, which mainly include CAFs, TAM, and T cells. ROS-relevant alternation in these cells contributes to inflammation, proliferation, angiogenesis, and metastasis. However, the accumulation of ROS upon a tolerant threshold causes mitochondrial and ER dysfunction, and even cell death. CAFs: cancer-associated fibroblasts; TAM: tumor-associated macrophage; AR: androgen receptor; ARE: androgen responsive element; SHBG: sex hormone-binding globulin.

Based on the diversified functions and interactions of ROS as well as a certain degree of understanding on aetiology of PCa, ROS have been identified to play critical roles in the pathogenesis of PCa. One characteristic of PCa cells that distinguishes them from normal cells is having higher ROS levels associated with upregulated key components of ROS producers and antioxidant enzymes/peptides. These components include ETC, NOXs, p66Shc, Nrf2, TRx, and GSH. A moderate level of ROS is required for the progression of PCa via ROS-dependent reduction-oxidation reactions and signaling pathways, such as genetic instability, epigenetics aberrations, AR signaling, and autophagy. Additionally, the oxidative microenvironment of PCa resulting from a group of various nonmalignant cells with large amounts of ROS, like CAFs, TAM, and T cells, provides favorable circumstances that contribute to drug resistance, metastasis, and immune evasion of PCa cells. However, elevated levels of ROS generated to toxic levels or exhaustion of the critical antioxidant system capacity would result in PCa cell death (Figure 2). ROS regulations represent a potential target for the treatment of PCa. Currently, given that ROS are also an “Achilles' heel” in tumors, two strategies have been developed [227]. The treatment with natural antioxidants is regarded as an essential focus on retarding PCa progression via quenching ROS and reducing oxidative stress. As such, the use of antioxidants significantly enhances the antitumor efficacy when synergistically combined with other therapeutics that induce cell death independent of oxidative stress. On the other hand, a further elevation in the ROS level mediated by ROS producing agents or those abrogating the inherent antioxidant system crosses the tolerable threshold, thus resulting in various types of cell death. In this regard, most of the currently available prostate cancer therapeutics are highly dependent on ROS-developed cytotoxicity. In summary, the results of this study indicate that ROS, as a common proliferative and apoptotic convergent point, regulate the biological behaviors subtly in terms of different cellular environments. However, it is necessary further to shed light on the exact mechanism of ROS influencing PCa.

## Figures and Tables

**Table 1:** Clinical studies conducted the chemoprevention of PCa by the antioxidants.

No.	Antioxidants	Mechanism	Major outcome	References
1	A–tocopherol	The downregulation of PSA levels	A–tocopherol slowed the progression of PCa patients with biochemical recurrence; Higher serum a-tocopherol at baseline improved PCa survival	[228]
2	A-carotene	A-carotene negatively regulate percent free PSA level, but not total PSA	A–carotene conferred a favorable prognosis after PCa recurrence.	[229]
3	Lycopene	Significant declines in serum PSA and markers of oxidative DNA damage; Prolongation of PSA doubting time	Lycopene was associated with a reduced risk of lethal PCa and enhanced the efficiency of radical prostatectomy.	[230, 231]
4	Vitamin D	Vitamin D slowed the rate of PSA increase	Vitamin D was beneficial to patients with asymptomatic progressive PCa; Vitamin D improved response rate and increased median survival time in patients taking docetaxel therapy.	[232, 233]
5	Selenium	Selenium regulated GPX1 to reduce lipid and hydrogen peroxides to water.	Selenium reduced PCa susceptibility and the risk of aggressive PCa.	[234–236]
6	Zinc	Inhibitions of metallothionein and NOX expression; Zinc served as a cofactor for the SOD enzyme.	Zinc improved survival only in men with early-stage cancers; Zinc modestly reduced the risk of high-grade disease	[237, 238]
7	Soy isoflavones	Soy isoflavones inhibited NF-*κ*B and HIF-1*α* up-regulated by radiotherapy.	Soy isoflavones sensitized PCa patients to the radiotherapy and mitigated normal tissue injury.	[239, 240]
8	Green tea catechins	The electron delocalization and free radical scavenging	Green tea catechins served as secondary chemoprevention of PCa and reduced PCa incidences of men diagnosed with HG-PIN.	[241, 242]
9	Resveratrol	Resveratrol diminished NOX activity and increased the expression of CAT and glutathione reductase; Resveratrol prolonged the doubling time for PSA.	Resveratrol decreased the risk of PCa in men with the SOD2 Ala/Ala genotype.	[243]

Abbreviations: PSA: prostate-specific antigen; GPX 1: glutathione peroxidase 1; NOX: NADPH oxidase; SOD: superoxide dismutase; NF-*κ*B: nuclear factor kappa-B; HIF-1*α*: hypoxia inducible factor-1*α*; HG-PIN: high-grade prostatic intraepithelial neoplasia; CAT: catalase.
